# Adapting Team-Based Learning for Medical Education: A Case Study with Scalable and Resource-Efficient Implementation

**DOI:** 10.1007/s40670-024-02246-y

**Published:** 2024-12-19

**Authors:** Michaela Jansen, Irina Kim Cavdar

**Affiliations:** 1 Department of Cell Physiology and Molecular Biophysics, Texas Tech University Health Sciences Center School of Medicine, Lubbock, TX 79430 USA; 2 Department of Medical Education, Texas Tech University Health Sciences Center School of Medicine, Lubbock, TX 79430 USA

**Keywords:** Team-based learning, Active learning, Implementation

## Abstract

**Purpose:**

Team-based learning (TBL) is a structured collaborative learning strategy where students are able to apply conceptual knowledge in small groups through a sequence of activities comprised of preparation, individual readiness assurance testing, teamwork, and team readiness assurance testing, typically followed by an application exercise. TBL has been gaining popularity in many education institutions and programs across the world and in the USA. This study marks the beginning of implementing TBL as a major active learning modality at Texas Tech University Health Sciences Center School of Medicine. Through this initiative, we present TBL as a versatile and adaptable method to improve students’ learning and examination outcomes.

**Methods:**

TBL sessions are conducted in three steps: pre-class preparation, in-class readiness assurance testing, and application-focused exercise. The present study used a modified or abbreviated format, consisting of pre-class preparation and in-class individual readiness assurance test (iRAT) and team readiness assurance test (tRAT) followed by immediate feedback. A Pilot Phase was used to engage early-adopter faculty and optimize session parameters, and an Implementation Phase was used for one organ system, with both phases in the pre-clerkship curriculum and a class size of 180 students. During the Pilot Phase, student participation was voluntary, whereas in the Implementation Phase, participation counted towards a selective session attendance requirement. Therefore, student numbers were significantly smaller during the Pilot Phase supporting gradual optimization of session organization.

**Results:**

iRAT, tRAT, and summative end of organ system section National Board of Medical Examiners (NBME) scores were analyzed. We find that participation in an increasing number of TBLs was associated with increased iRAT scores and a decreased performance gap between highest and lowest performers, where the increase in the scores of lowest performers was more substantial than the increase in the scores of highest performers. NBME score analysis showed that TBL participation increased examination performance by an average of 2.4% per TBL session attended.

**Conclusions:**

We present an implementation strategy for TBL sessions using a two-phased approach. Our process implementation provides a clear roadmap for other health professions or medical schools to implement TBL format sessions in their specific educational context. Importantly, the unique, abbreviated TBL format presented here facilitates implementation and adaptation. Observed learning strategies during tRAT that have been demonstrated to be effective include elaboration, dual coding, specific examples, interleaving, and retrieval practices. Overall, the results indicate a positive impact of TBL participation on final summative exam scores.

**Supplementary Information:**

The online version contains supplementary material available at 10.1007/s40670-024-02246-y.

## Introduction

### Origins of TBL and Its Beginning in Medical Education

Over 900 years ago, with the appearance of universities in Western Europe, lecturing became the predominant mode of instruction. In the early 1900s, proponents of active learning challenged this traditional teaching method, arguing that in a rapidly changing society, the method in which the teacher dispenses packaged knowledge to the students is inadequate for the present and future needs of students. They advocated for education programs based on cognitive learning and the relationship of school to society [1]. Since then, active learning has had many descriptions, including discovery, student-centered, cooperative learning, the project method, democratic education, learning by doing, and progressive education.

Similarly, over the past 15 to 20 years, preclinical medical curricula have changed significantly in pedagogy. This change was preceded by 80 years of medical education in the USA that was mostly delivered via didactic lecture. Recent changes have been driven by a growing number of educators [1–3]. Based on increasing evidence that active learning strategies yield desirable learning outcomes, as measured by test scores, and other beneficial learner skills, such as communication, many medical schools have replaced part of their lectures in their preclinical curricula in favor of active learning instructional methods [3]. One such method is team-based learning (TBL). TBL was first developed and described by Larry Michaelsen as “an active learning and small group instructional strategy that provides students with opportunities to apply conceptual knowledge through a sequence of activities that includes individual work, team work, and immediate feedback” [4]. Michaelsen then implemented TBL for small-group large classroom settings as class sizes in the business school environment increased in the early 1990s. TBL involves individual student preparation before the TBL session and the TBL session itself (Table 1). The TBL session comprises two multiple-choice tests and a subsequent application exercise that typically contains a discussion of vignettes or case studies led by a facilitator who is an expert in the topic and whose role is to foster the effectiveness of group work [5]. The multiple-choice tests are an individual readiness assurance test (iRAT) that students first answer individually, and subsequently a team readiness assurance test (tRAT) that students complete as groups of 5 to 7 students by discussing the question and agreeing on the best answer. The tRAT is followed by a large classroom debrief of particularly challenging questions or the discussion of an application problem. In undergraduate medical education, TBL was first introduced as “team learning” within a medical physiology course at Baylor College of Medicine by Charles Seidel and Boyd Richards in 2001 with the objective “to try out an instructional method, team learning (TL), to achieve levels of active learning and group skills similar to [problem-based learning] PBL but in a lecture setting with a single instructor” [6]. TBL is different from other active learning approaches that involve small-group approaches in that there is no need for multiple faculty (up to 1:200 students) or rooms.

**Table 1:**
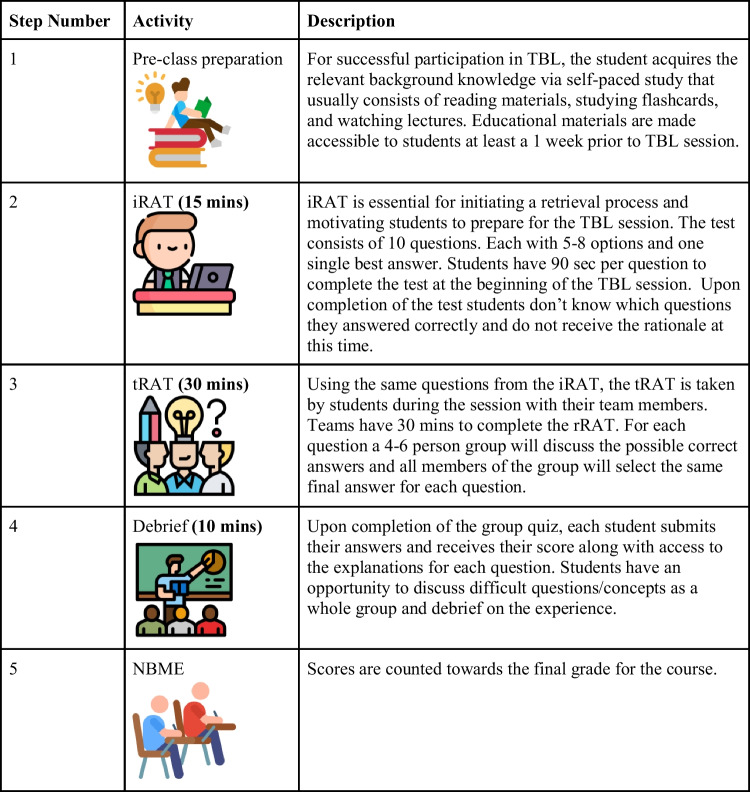
Steps for abbreviated TBL sessions optimized through Pilot and Implementation Phases at TTUHSC SOM (each TBL class is 1 h)

### Cognitive Psychology Mechanisms Enabling Knowledge Reconsolidation During TBL

Student preparation for a TBL session involves initial learning and subsequent stabilization of the initially labile memory by a process called memory consolidation [7]. During the TBL session itself, different psychological mechanisms have been proposed to foster knowledge reconsolidation at different stages of the session [8]. Retrieval practice, peer elaboration, feedback, and transfer (ability to make connections and apply learnt concepts to other situations) have been suggested as the basic psychological mechanisms and integral parts of TBL. Retrieval practice occurs during the iRAT, peer elaboration during the tRAT, feedback during the tRAT and its debrief, and transfer during the application exercise. TBL’s emphasis on retrieval practice and the amount of time devoted to application exercises has been highlighted as unique amongst well-known active learning strategies. Repeated retrieval allows students to reactive what they learnt previously and develop extremely stable structures of knowledge [9–11]. Repeated testing in TBL enhances knowledge retention, a phenomenon called testing effect [12], leading to a higher long-term examination performance.

### Challenges of TBL and Aim of This Study

Although TBL has been gaining popularity in many education institutions and programs since 2000, not all US medical schools have adopted this approach. Factors related to faculty, student, course, and institutional environments were associated with changes in TBL use. For example, a longitudinal study at 10 medical schools, conducted over 2 years, found that TBL use was continued in 9 out of the 10 original schools. At the start of the study, 32 courses used TBL. After 2 years, TBL use was continued in 19 and discontinued in 13 courses. The main reason for discontinuation was the introduction of TBL into 18 new courses, where it was the primary teaching method within integrated courses. TBL use was discontinued completely at 1 school due to the departure of a key faculty partner [13].

With the present study, we provide a concrete example of implementing TBL sessions using a two-phased approach at a US medical school, Texas Tech University Health Sciences Center School of Medicine (TTUHSC SOM), within our integrated, preclinical curriculum. By showcasing our unique abbreviated format, we aim to inspire institutions predominantly using lectures to consider the integration of TBL sessions by demonstrating the versatility and scalability of this active learning strategy. Additionally, we present optimized protocols developed during this project to support seamless adoption by other medical schools.

Key challenges to TBL implementation including securing faculty buy-in and ensuring session consistency were addressed through our approach. During the Pilot Phase, we engaged early-adopter faculty to volunteer as TBL facilitators, allowing us to refine the format before broader implementation. Our streamlined approach combined with the unique and abbreviated TBL format consisting of pre-class preparation, readiness testing (iRAT and tRAT), and feedback simplified both preparation and delivery. By providing faculty with clear session structure guides, we ensured consistent, high-quality TBL experiences that can serve as a model for other institutions.

## Methods

### Ethical Approval of Study

The study was approved by the TTUHSC Quality Improvement Review Board (QI-23127).

### Abbreviated TBL Format in This Study

TBLs are traditionally conducted in three steps: pre-class preparation, in-class readiness assurance testing with iRAT and tRAT, and application-focused exercises. However, for the present study, we used a unique, abbreviated format consisting of presession preparation and in-class sessions with iRAT and tRAT, while omitting the group-based application exercises. This adjustment was made to fit the sessions into a 1-h time slot, which we believed would be better received by both students and faculty. Additionally, this format significantly simplified faculty preparation, reducing the workload associated with designing and facilitating application exercises. By streamlining the sessions, we aimed to increase participation rates and acceptance of the TBL format while still maintaining the core benefits of readiness assurance and collaborative learning. TBL sessions were concluded with a group discussion and faculty feedback following the tRAT. The in-class portion of each TBL session was 1 h long.

### TBL Setting

**Fig. 1:**
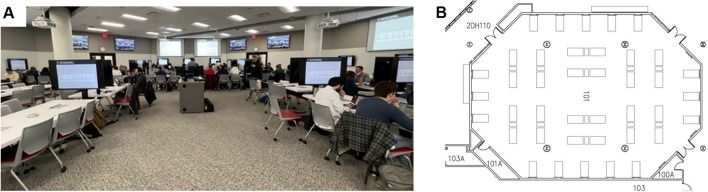
Large classroom with small-group arrangement. **A** Photo of a typical TBL session. **B** Arrangement of 40 tables with 6 seats, each, in the classroom. In addition to instructor projection monitors on the walls, each table has a monitor to which learners sitting at the table can project from their personal devices

TBL sessions were held in Pilot (Fall of 2022) and Implementation Phase (Spring of 2023) settings for pre-clerkship first- and second-year medical students. The Pilot Phase TBL sessions were alongside the Organ Systems 4 course for second-year students that covers neurosciences, behavioral sciences, and reproductive systems. Second-year students were chosen for the Pilot Phase based on their familiarity with the multiple-choice question (MCQ) platform (UWorld) used for the session. The session content was focused on first-year content. The Implementation Phase sessions were held as integral sessions within the Organ Systems 1 course for first-year students. These students were novices in the use of the MCQ platform. TBL sessions were held in a large classroom with 40 six-seat tables (Fig. 1). Students were instructed to sit in groups of 6. Students were free to choose their group and table. Each group table was associated with a screen at its side, to which students could project their personal device’s screen for small-group discussions. Additional large screens on the sides of the classroom were used to display instructions and timelines for the TBL session related to accessing the iRAT and tRAT (see Appendix A).

### TBL Session Parameter Optimization

The Pilot Phase was conducted with second-year students, and this phase was used to optimize procedures for administering the TBLs. This phase included eight 1-h TBL sessions, each targeting a predefined content area from first-year foundation or organ systems courses. TBL content areas and dates were announced for each TBL session at least 1 week in advance. Each in-class session consisted of a 5-min introduction, 15-min iRAT, and 30-min tRAT. Given this timing, we found that the sessions were most efficient with ten items for the iRAT/tRAT. For the iRAT, students individually completed a ten-item, timed MCQ test online using the UWorld Learning Platform on their personal devices (laptop or iPad). Subsequently, each table or team completed a duplicate team version of the iRAT as tRAT, selecting the single best answer for each item based on group elaboration and discussion. Each student submitted the tRAT version after selecting answers based on group discussion. Faculty explained concepts for the most difficult questions after tRATs were submitted.

### TBL Implementation

The Implementation Phase was conducted during the cardiovascular module of the Organ System 1 course. Five TBLs were scheduled over 5 weeks. The goal was to analyze participation rate and determine whether participation in TBL sessions correlated with performance on the final summative exam, a customized National Board of Medical Examiners (NBME) examination. Participation was monitored by using UWorld scores and counted towards an attendance requirement of 75% of all selective sessions within the course. Similar to the Pilot Phase, immediate feedback and material for review were provided by releasing the rationales in Uworld, after the tRAT quizzes were submitted.

### Unique Adaptations

Our TBL implementation satisfied principles of TBL and followed the core of the TBL format as described by Michaelsen [3, 14]. However, it possessed unique aspects: The primary adaptation from the standard TBL format is our unique, abbreviated approach, which omits the application exercise typically included in TBL sessions. This streamlined format allows us to conduct sessions within a 60-min timeframe, which we believe contributes to greater acceptance from both faculty and student compared to the longer, standard TBL format. Other unique aspects include that student participation was voluntary, and teams were self-assembled by students and not fixed. To ensure uniformity between different TBL sessions, instructors received step-by-step guides (Appendix A) for planning and facilitating sessions, including test parameters for iRAT and tRAT using UWorld, team size, and slide presentations to facilitate technical aspects of sessions. Students received a brief introduction on strategies for effective learning using publicly available resources (https://www.learningscientists.org/downloadable-materials) and an overview for what to expect for a session of the abbreviated TBL format (Appendix B). This is in contrast to instructions provided in the TBL Guide, which indicates that presession faculty and student instructions for TBL are not needed [3]. The iRAT and tRAT scores did not count towards a final course grade, eliminating the need for appeals, which is highlighted in the TBL Guide [14]. Students’ self-motivation to master the material, selective attendance requirement, and understanding of the potential impact of TBL on their final grades could be interpreted as incentives for students to learn materials beforehand, attend sessions, and contribute to collaborative team discussions during the tRAT and debrief.

### Data Analysis

In the Pilot Phase, we analyzed participation rates when attendance was voluntary and compared performance on iRATs and tRATs. In the Implementation Phase, we assessed participation rate when attendance counted towards an attendance requirement and similarly compared iRAT and tRAT performance. We used the Kruskal–Wallis test followed by uncorrected Dunn’s test (GraphPad Prism) to compare iRAT and tRAT scores for each session. Additionally, we compared averaged iRAT vs averaged tRAT scores in the Pilot and Implementation Phases using an unpaired two-tailed *t*-test. For the Implementation Phase, we assessed for the potential association between participation in an increasing number of TBL sessions and both iRAT scores and performance on the final summative National Board of Medical Examiners (NBME) exam.

## Results

### Participation Rate

**Fig. 2:**
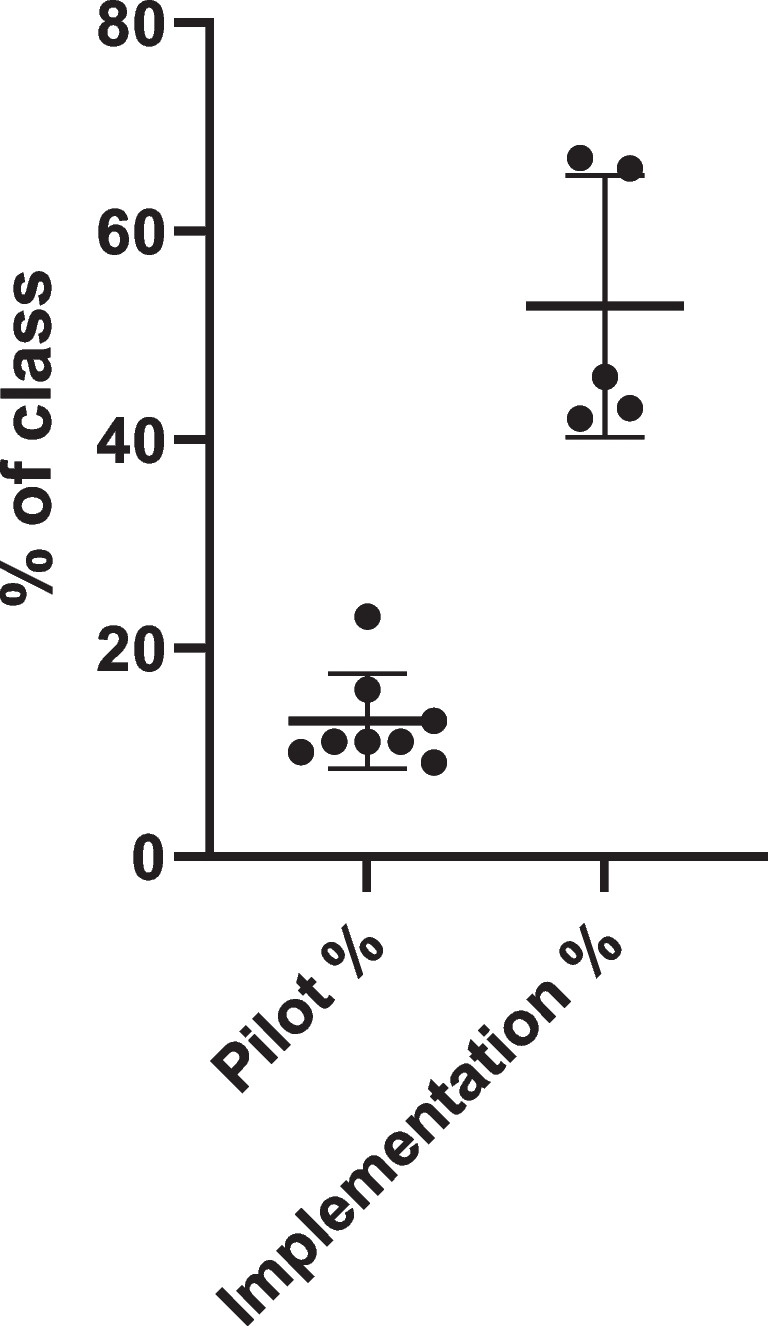
Participation rate for the pilot and implementation phase. During the pilot phase, attendance at the TBL sessions did not count towards an attendance requirement, whereas during the implementation phase, it counted towards a selectives attendance requirement

Each medical school class has about 180 students. A participation rate of 13.0 ± 4.6% (24 ± 8 learners) was observed for the pilot phase when TBL sessions were first introduced and student attendance at the sessions was entirely voluntary and did not count for an attendance requirement. Participation rate significantly increased to 52.8 ± 12.6% (96 ± 23 learners) when it counted towards an attendance requirement during the implementation phase (Table 2, Fig. 2).

**Table 2:** Data for individual TBL session in the pilot and implementation phases. Participation rate is provided in number (#) of students participating and the respective percentage (%) of the class, mean iRAT and tRAT scores are provided with their respective SD values, the difference between mean tRAT and mean iRAT scores is provided, and the *p*-value for each session indicated (Kruskal–Wallis test, uncorrected Dunn’s test)

TBL session	# students	%	Mean iRAT	SD	mean tRAT	SD	Mean tRAT − mean iRAT	*p*
Pilot 1	30	16.4%	54.4	19.0	88.0	11.6	33.6	< 0.0001
Pilot 2	42	23.0%	50.0	19.0	90.7	7.5	40.7	< 0.0001
Pilot 3	20	10.9%	53.5	19.3	94.2	17.1	40.7	< 0.0001
Pilot 4	24	13.1%	55.0	21.1	92.6	8.6	37.6	< 0.0001
Pilot 5	16	8.7%	48.1	14.0	74.9	5.6	26.8	0.0199
Pilot 6	21	11.5%	45.2	18.1	96.0	7.4	50.8	< 0.0001
Pilot 7	19	10.4%	42.8	19.0	84.7	17.4	42.0	< 0.0001
Pilot 8	20	10.9%	58.5	23.2	98.5	3.7	40.0	< 0.0001
Implem. 1	123	67.2%	49.4	17.3	92.5	9.7	43.2	< 0.0001
Implem. 2	120	65.6%	53.3	17.3	89.8	14.2	36.6	< 0.0001
Implem. 3	84	45.9%	48.7	18.0	78.6	19.0	29.9	< 0.0001
Implem. 4	76	41.5%	58.3	22.2	91.0	15.4	32.7	< 0.0001
Implem. 5	78	42.6%	48.1	20.7	88.7	17.4	40.6	< 0.0001

### Readiness Assessment Test Comparison

The mean scores for iRATs and tRATs in both the Pilot Phase and the Implementation Phase are summarized in Table 2. For each TBL session in both the Pilot and Implementation Phases, the iRAT scores were lower than the tRAT scores and statistically different (Kruskal–Wallis test, uncorrected Dunn’s test). Therefore, as expected, by session averaged tRAT scores were significantly higher as compared to by session averaged iRAT scores, with *p*-values of *p* = 0.0004 and *p* = 0.0131 for both the pilot and implementation phases, respectively (Table 3). The tRAT − iRAT differences for the pilot and implementation phases were not significantly different (unpaired *t*-test, two-tailed).

**Table 3:** Summary data for the pilot and implementation phases

	# TBL sessions	Participation rate	iRAT	tRAT	tRAT − iRAT	*p*
Pilot	8	13.1 ± 4.6% (24 ± 8.4 learners)	50.9 ± 5.4	89.9 ± 7.5	39.0	0.0004
Implem	5	52.2 ± 13.2% (96 ± 23.3 learners)	51.6 ± 4.3	88.1 ± 5.5	36.6	0.0131

### Participation in an Increased Number of Sessions and Performance Indicators

**Fig. 3:**
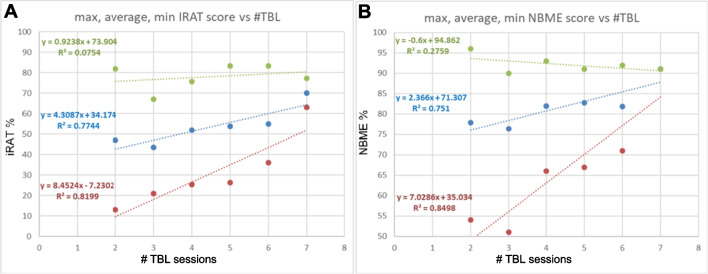
Participation in increasing number of TBL sessions and performance indicators. The number of TBL sessions individual students participated in is depicted on the horizontal axis from two sessions to seven sessions. **A** iRAT score dependence on number of sessions attended. iRAT scores are plotted against the number of sessions color-coded by lowest (red), mean (blue), and highest (green) scores. **B** NBME score dependence on number of sessions attended with colored data points and trendlines as in **A**. Note that the average performance and lowest performance trendlines have positive slopes indicative of participation in an increased number of sessions associated with increased performance

We also investigated whether there was an effect of participating in an increasing number of TBLs on iRAT performance and/or on NBME course exam performance. The results indicate that participation in an increasing number of TBLs was associated with increased iRAT scores with an average of 4.3% increase in iRAT score per additionally attended TBL and a decreasing performance gap between highest and lowest performers (Fig. 3), where the increase in the scores of lowest performers contributed more substantially to this phenomenon than the increase in the scores of highest performers. We infer that TBL allowed lower performers to develop and evolve their learning skills or comprehension to become more efficient learners or test takers. Looking at iRAT scores, students at both ends of the spectrum, highest and lowest performers, benefited from group discussion, the latter most probably because explaining concepts to others reinforces one’s own knowledge [2].

At the end of the course in which the Implementation Phase TBL sessions were scheduled, a summative National Board of Medical Examiners (NBME) examination was administered. The results reported here indicate that NBME examination performance increased by an average of 2.4% per TBL session attended.

Overall, the results indicate that the students who participated in more TBL sessions had higher final grades as measured by NBME scores. This supports the hypothesis that group-based activities associated with TBL sessions encourage the reconsolidation of knowledge that students acquired previously [9], leading to better examination outcomes. Alternative explanations may be increased preparation for TBLs or acquisition of new information from peers during TBLs. Observed learning strategies during tRAT that have been demonstrated to be effective include elaboration, dual coding, specific examples, interleaving, and retrieval practice [15–20]. Of these strategies, it is worth highlighting retrieval practice and feedback. The amount of effort to retrieve material from memory moderates the learning effects of explaining to others, and the degree of elaboration during retrieval practice positively predicts the outcomes of learning [21]. Meanwhile, feedback is another potent mechanism to increase student learning and performance [22, 23]. Providing feedback on test responses has been shown to decrease levels of anxiety via freeing working memory and improving focus during the actual exam. Therefore, higher student performance may partially be explained by TBL elements, such as tRAT and timely feedback, that mitigate anxiety and facilitate a sense of control during summative exams, in this case a customized NBME [16].

## Discussion

### Feasibility and Transferability

This study marks the beginning of increasing the number of TBL sessions throughout the pre-clerkship curriculum as a major mode of active learning at TTUHSC SOM. The abbreviated TBL format presented here as an adaptable method aimed at enhancing student learning and examination outcomes. Several factors were critical to successfully implementing TBL in a medical school setting: The institutional culture supported instructional innovation, and students were receptive to the trial-and-error nature of a new strategy. The Pilot Phase of this study involved early-adopter faculty across different courses in the curriculum, who volunteered to host single, non-mandatory sessions. Additionally, a comparatively smaller number of students during this pilot phase allowed for feasibility testing and optimization of session parameters (e.g., quiz release times, number of items on quiz, time allocation for iRAT and tRAT).

The course leadership faculty for the Organ System 1 course in the Implementation Phase were also involved in the Pilot Phase, ensuring continuity. Faculty initiating TBL sessions were receptive to ongoing informal student feedback, leading to adjustments in session parameters as needed, particularly for larger numbers of learners. Normally, organizing a TBL module is resource intensive, but integration into the curriculum here was greatly facilitated by leveraging a question bank with a large number of MCQs iRAT or tRAT assembly, and access to a large classroom that accomodates small-group arrangements. We used a classroom with 40 six-seat tables (Fig. 1), each with an individual screen and large wall screens to guide the overall session. Students were observed in lively discussions with some groups using the available technology in this ideal facility for a small-group large classroom setting, and other groups using their personal tablets or laptops or paper to draw, share, and explain their answers. While our specific classroom setup and technology were ideal, any large room that supports small groups to interact and discuss and potentially spread out to reduce (noise) interference across groups would seem appropriate for this setting. Some well-attended sessions can get quite loud. In these situations, sessions can be scheduled in duplicate to split a large class size in half.

### Scholastic and Non-scholastic Benefits of TBL

TBL sessions provide an opportunity for students to assess gaps in their understanding and share their concerns with peers and faculty. The peer pressure and natural deadlines for completing work inherent in TBL encourage students to avoid procrastination and engage in self-testing. In the TBL context, students may also realize that they are not the only student not knowing an answer and that even when all team members got an item on the iRAT wrong, they can still as a group come to the correct answer. TBL also reinforced the value of testing to identify weaknesses thereby supporting a growth mindset. We hypothesize that these benefits could have led to the observed higher NBME performance for students who participated in TBL sessions more often [24].

As importantly, we observed that students were learning how to work, interact, and collaborate in a team during the TBL sessions. Thus, TBL can help with integration of knowledge, while developing interpersonal and collaborative skills. For example, the development of communication skills was first encouraged during team test discussions in TBL and then by seeking feedback that allowed students to generate a scholarly formulated argument or a question. Finally, the students engaged in intra-team communication during tRAT. Working together in self-determined teams encouraged friendships that became instrumental in forming informal, outside-of-classroom, small study groups. These sessions also enabled students to develop closer collegial connections with their faculty that would be more difficult to achieve in a large lecture classroom setting. Studies have shown that developing such relationships with peers and faculty can be a safeguard against dropout [25].

In the medical field or the healthcare sector in general, there is a demand for students who can coordinate with others, engage in critical thinking, and demonstrate cognitive flexibility, emotional intelligence, complex problem-solving, judgment, and decision-making [26]. Several studies have shown that TBL, in contrast to the traditional one-way lecture format, meets these competency-based challenges [5, 27–31]. Thus, future iterations of the optimized TBL sessions will be consistently implemented within all Organ System Courses of the curriculum.

### Resource Efficiency and Scalability

In this study, attendance and participation played a significant role in the effectiveness of TBL sessions. To respect faculty and students’ autonomy, sessions were offered as selectives, allowing learners to choose based on their needs. Although this study is based on a unique abbreviated adaptation of TBL, our results are consistent with previous studies demonstrating TBL’s positive impact on exam performance [30]. Additionally, similarly to traditional TBL, our abbreviated TBL still allowed learners to practice other skills essential to success in increasingly complex healthcare systems, like communication and collaboration. The effects of TBL reported here are subject to important qualifications, however. For example, further investigation in causality needs to be conducted to elucidate the relationship between higher scores on NBME and participation in TBLs. We entertain the possibility that high-performing students attended more TBLs since they had more time, while low-performing students chose self-study. We also hypothesize that high-performing students chose participation in TBLs for other non-scholastic benefits outlined earlier.

TBL proved resource efficient in digitally enabled classroom settings, reducing the amount of time to prepare compared to traditional lectures. Facilitators could easily content and questions to be discussed in class, for example, based on misconceptions revealed in small-group discussions.

Since most of the learning occurs in the form of discussions and feedback sessions in class, unnecessary detail and extensive preparation are minimized. Finally, TBL is scalable to much larger student-to-faculty ratios of 200 to 1 [32] and can be facilitated in large classroom settings. The quality of the discussion during completion of the tRAT depends on students’ motivation for in-depth dissection of each question. If students are free to leave the session early, they may rush to just complete the TBL. An additional consideration may be the impact of expertise of the facilitating faculty on the success of the TBL sessions. In several instances, faculty who led a session were not content experts for the respective TBL session content but highly supportive of the TBL format. The feedback in some sessions was limited to UWorld rationales. We did not find that this impacted the student experience for these sessions. With a more formal inclusion of the TBL format in our curriculum, TBL sessions will nonetheless be led by content experts.

TBL’s scalability allows for a large group of students to participate in small-group learning experiences, with a small number of faculty facilitators. Thus, introduction of TBL presents a resource-saving option in terms of required faculty. We believe that the benefits and the positive outcomes will be further multiplied as more TBL-specific technologies start to emerge aiding in gathering detailed data and further optimization of TBL sessions. In addition, we infer that institutions that adopt TBL or similar active learning strategies consistently will see improvements in student performance and engagement.

### Challenges and Adaptations

Transforming pedagogical practice is challenging, particularly for basic science and clinical science medical faculty with limited prior exposure to TBL pedagogical theory and training. Allowing for institution-specific adaptations and gradual implementation of TBL, as described here, may ease the transition from traditional lecture-centered approaches to active learning through TBL.

### Strengths and Limitations

The study acknowledges several limitations. The small sample size of participating students during the pilot phase (24 students) may limit the generalizability of findings for this small cohort. Additionally, the study only evaluated short-term impacts of TBL, lacking longitudinal data to assess its effectiveness over time. TBL was only introduced and investigated within one organ section within a single course for the present study. Since participation was partially voluntary, the self-selection of motivated students may have biased the results. Inconsistencies in team composition across sessions could also have influenced outcomes due to varying team dynamics. We did neither control nor record team composition. The study lacks formal qualitative feedback on student motivation, which could have provided deeper insights. Additionally, not all faculty facilitators were content experts, potentially impacting the consistency of TBL sessions. Finally, familiarity with the same questions in iRAT and tRAT assessments might have inflated scores, affecting the measure of genuine learning improvements.

## Conclusion

The study highlights the feasibility and positive impact of an abbreviated TBL format in enhancing student learning and engagement in a medical education context. By leveraging institutional support, iterative feedback, and adaptation of session parameters, we successfully integrated TBL into the pre-clerkship curriculum. The observed improvements in both scholastic and non-scholastic domains demonstrate the potential of TBL to address competency-based educational needs in the medical field.

While TBL offers numerous benefits, such as resource efficiency and scalability, further research is required to establish causality between TBL participation and improved exam performance. Additionally, future iterations should include content experts to maximize the educational impact. We anticipate that institutions embracing TBL methodologies will experience enhanced student outcomes and engagement, ultimately contributing to the development of healthcare professionals equipped with essential collaborative and critical thinking skills.

## Supplementary Information

Below is the link to the electronic supplementary material.

Supplementary file1 (DOCX 23 KB)

Supplementary file2 (DOCX 24 KB)

Supplementary file3 (DOCX 68 KB)

## Data Availability

The datasets generated during and/or analysed during the current study are available from the corresponding author on reasonable request.
