# Supplementary material for: Adapting Team-Based Learning for Medical Education: A Case Study with Scalable and Resource-Efficient Implementation
Source: Med Sci Educ. 2024 Dec 19;35(2):883–92. doi: 10.1007/s40670-024-02246-y (PMC12058633; doi:10.1007/s40670-024-02246-y)
Supplement: Supplementary file 1 — Supplementary file1 (DOCX 23 KB) [file 40670_2024_2246_MOESM1_ESM.docx]

## TBL Session General Outline:

TBL was first developed and described by Larry Michaelsen as “an active learning and small group instructional strategy that provides students with opportunities to apply conceptual knowledge through a sequence of activities that includes individual work, team work, and immediate feedback”.

TBL involves individual student preparation before the TBL session and the TBL session itself. **The TBL session comprises of two multiple-choice tests and a subsequent application exercise** that is typically a discussion of vignettes or case studies led by a facilitator who is an expert in the topic and whose role is to foster the effectiveness of group work.

The multiple-choice tests are an individual readiness assurance test (iRAT) that students first answer individually, and subsequently a team readiness assurance test (tRAT) that students complete as groups of 5 to 7 students by discussing the question and agreeing on the best answer. If we were to follow the strict definition of a TBL, the tRAT would be followed by an application exercise which would require another hour. Therefore, we conduct mostly abbreviated TBL sessions at this point, where the tRAT is followed by a large classroom debrief of particularly challenging questions.

**A TBL session will involve all of the following**

1. iRAT (15 minutes)
2. tRAT (30 minutes)
3. debrief of challenging questions with faculty lead (10 minutes)

## Before Session:

- Sign into Uworld
- Learning Platform
- On the top right you must select the correct class
  - Implementation:
    - Choose correct class, Co 2027 etc.
- Click assignments (either in left navigation bar or top tab)
- Click blue button “Create Assignment”
  - Step 1
  - Check subjects and systems to filter for your topic
  - Apply
  - Click on the eye and look at questions/switch on rationales as desired
  - Add questions to selection (you can always trim down further later)
  - Select 10 questions
  - Create assignment
  - Please do not safe the assignment as draft but rather go all the way to assign since sometimes Uworld temporarily seems to lose drafts.
- Create assignment in Uworld assigned to class
  - Name: TBL_iRAT_*topic* (*your topic*)
  - Settings:
    - Answer all questions
    - **Score and Explanation later via gradebook (so that students do NOT see score or in/correct answers after iRAT submission)**
    - 0 retakes allowed
    - Randomize the set
    - Assigned: date of session, start time of session
    - Due: date of session, 30 minutes past start time of session
    - Time limit: timed (will be 15 minutes)
- Duplicate quiz
  - Name: TBL_tRAT_*topic*
  - Settings:
    - Answer all questions
    - Explanation After assignment is submitted
    - 0 retakes allowed
    - Don’t randomize
    - Assigned: date of session, 20 minutes past start time of session
    - Due: date of session, time end of session
    - Time limit: custom (set to 30 minutes)

## Session:

- Classroom location is small group large classroom [room #]
- Direct students to sit in groups of 6.
- See separate document that will be distributed to students.
- See separate ppt to manage session (edit as desired)
- Make sure students do the individual quiz first and the group quiz second (the start times should help with that)
