# Supplementary material for: Adapting Team-Based Learning for Medical Education: A Case Study with Scalable and Resource-Efficient Implementation
Source: Med Sci Educ. 2024 Dec 19;35(2):883–92. doi: 10.1007/s40670-024-02246-y (PMC12058633; doi:10.1007/s40670-024-02246-y)
Supplement: Supplementary file 2 — Supplementary file2 (DOCX 24 KB) [file 40670_2024_2246_MOESM2_ESM.docx]

# Team-Based Learning (TBL) Sessions

## Overview:

This session will provide an opportunity to revisit and review content covered in the respective section of the block by answering a set of Step 1-like questions selected from the UWorld Question Bank.

## Effective Learning Strategies:

To review effective learning strategies please review: <https://www.learningscientists.org/faq>

## Goals:

1. Review respective content relevant to Step 1
2. Utilize clinical reasoning skills to answer a series of vignette-style questions
3. Collaborate within a team to select the single best answer to each question

Format:

This session will use a standard Team-Based Learning format as follows:

| xx:00 | Select a seat at one of the tables in room #. This should be no more than 6 people per table. Please fill in the spaces so that tables are full. |
| --- | --- |
| xx:05 | Follow instructions for attendance taking. |
| xx:10 | Begin a short quiz on your personal UWorld Questions Bank account. The quiz will be named “TBL_iRAT” and consist of 10-15 questions. You will have 90 seconds per question to complete the quiz. |
| xx:25 | Submission of iRAT. You will not know which questions you answered correctly and you will not receive rationales for these questions at this time. |
| xx:25 | Brief review of experience thus far and preparation for group work. |
| xx:30 | As a group at each table, each member will open a duplicate version of the quiz named “TBL_tRAT”. For each question the group will discuss all answer choices. All members of the group ideally will agree on the same single best answer. You will have 2-3 minutes to answer each question. |
| xx:50 | Upon completion of the group quiz, each student will submit their answers and will receive their score along with access to the rationales for each question. |
| xx:50 | Whole group discussion of difficult questions/concepts and debrief on the experience. |
