# Supplementary material for: Adapting Team-Based Learning for Medical Education: A Case Study with Scalable and Resource-Efficient Implementation
Source: Med Sci Educ. 2024 Dec 19;35(2):883–92. doi: 10.1007/s40670-024-02246-y (PMC12058633; doi:10.1007/s40670-024-02246-y)

Supplementary Material

Figure S1: Score distributions for the individual readiness assurance tests and team readiness assurance tests. A) Pilot phase including eight different sessions, and B) Implementation phase including five different sessions. The score difference between each iRAT and tRAT scores for each session was assessed using Kruskal-Wallis test with uncorrected Dunn’s test. P-values are denoted as **** p<0.0001 and * p<0.03.


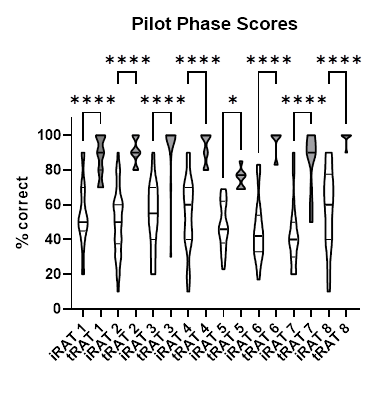

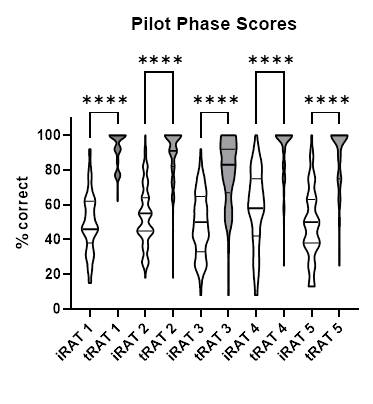


**B**

**A**

Figure S2: Mean iRAT and tRAT scores for all pilot and implementation phase sessions. Statistical difference was assessed using unpaired t-test, with p-values denoted as *** p=0.0004 and * p=0.0131.


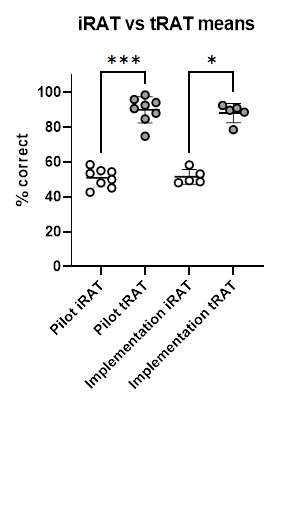

Supplement: Supplementary file 3 — Supplementary file3 (DOCX 68 KB) [file 40670_2024_2246_MOESM3_ESM.docx]
